# 2267 Radiofrequency renal denervation attenuates kidney fibrosis in spontaneously hypertensive rats

**DOI:** 10.1017/cts.2018.113

**Published:** 2018-11-21

**Authors:** Juan Gao, Ian B. Denys, Luis Del Valle, Mihran V. Naljayan, Daniel R. Kapusta

**Affiliations:** 1 Department of Pharmacology and Cardiovascular Center of Excellence, LSUHSC; 2 Department of Pathology and Stanley S. Scott Cancer Center, LSUHSC; 3 Department of Nephrology, LSUHSC

## Abstract

OBJECTIVES/SPECIFIC AIMS: The goal of this study was to investigate whether RF-RDN attenuates renal fibrosis and inflammation in SHR with established hypertension. METHODS/STUDY POPULATION: Twenty-two-week-old SHR received bilateral RF-RDN or Sham-RDN (Biosense Webster Stockert 70 generator and RF-probe). Four weeks later, SHR were sacrificed and paraffin sections of kidneys were stained for fibrosis by Masson’s trichrome staining. Kidney tissue were homogenized for measurement of cytokines levels by ELISA. RESULTS/ANTICIPATED RESULTS: The results showed that Sham-RDN treated SHR had extensive fibrosis as demonstrated by moderate thickening of Bowman’s capsule, collagen deposition in glomerulus, extensive tubulointerstitial fibrosis, and segmental glomerulosclerosis. In contrast, RF-RDN significantly reduced each of these pathological components of fibrosis in kidney cortex and medulla as compared with Sham-RDN treated kidneys. In other studies, RF-RDN decreased B cells, CD4+ T cells, and CD8+ T cells in the kidney of SHR as measured by flow cytometry. Meanwhile, kidney tissue levels of IL-17, INF-γ, MIP-3a, TNF-α, and TGF-β were decreased as compared with respective levels in Sham-RDN. DISCUSSION/SIGNIFICANCE OF IMPACT: Together, these findings demonstrate that removal of the influence of heightened renal sympathetic activity by RF-RDN decreases kidney inflammatory markers and attenuates renal fibrosis in hypertensive SHR.

